# Supplementary material for: How is hygiene behaviour affected by conflict and displacement? A qualitative case study in Northern Iraq
Source: PLoS One. 2022 Mar 3;17(3):e0264434. doi: 10.1371/journal.pone.0264434 (PMC8893612; doi:10.1371/journal.pone.0264434)
Supplement: S2 Appendix — (DOCX) [file pone.0264434.s002.docx]

Supplementary Materials – 2

Table 1: Purpose, description and sample size for each of the methods done within group discussions

| **Method** | **Description** | **Purpose** | **Origins and prior use** | **Sample size** |
| --- | --- | --- | --- | --- |
| Problem free-listing and categorisation | Ask participants to list the things they worry about most in their day to day lives since the crisis. Each worry is written down on a separate small piece of paper. Participants list as many things as come to mind. If not mentioned ask if the following things are challenges for them: ‘I often feel dirty and am not always able to wash my hands’, ‘It is hard to keep my home clean’, ‘I worry about the bathroom being dirty’. Explain that you would like them to sort these problems and their own problems into categories and give each category a name. Note where the hygiene related challenges are classified. The title of this category will be used for the individual free listing and ranking of problems (see Table 3). | This method is the first part of a two stage process to understand hygiene-related challenges in crisis contexts. This stage of the process aimed to generate an emic understanding of the challenges people faced as a consequence of the crisis and the prompts ensured that hygiene-related challenges were located and classified within this. The secondary purpose of this method was to define the ‘domain name’ under which people classified challenges relating to handwashing. This locally defined domain would then be used in the second part of the method (described in table 3) | Free-listing and categorisation are commonly used in qualitative research including work in the WASH sector. The two step process used in this research replicated a process outlined by Quinlan (1). | 2 focus groups (1 with men one with women) |
| Risk scaling | Participants are asked about the health issues that they are most concerned about. If diarrhoea is not mentioned this is brought up and participants are asked about their concern about diarrhoea in relation to other health concerns. Participants are then asked to define diarrhoea in terms of symptoms and causes so that this definition is clear for the rest of the session. Participants are introduced to a colour-coded, 5-point Likert scale which ranges from very likely to very unlikely. They are asked questions about their perceived vulnerability and susceptibility to diarrhoea and their perceived relative risk in comparison to other people and as a result of their current predicament. Individually participants select their perceived risk on the scale and then discuss differences. | This method aims to understand how the perceived risk of diarrhoea and or cholera differs among different population subgroups, at different stages of an emergency and in different types of settings. The group discussion format is designed to create debate and discussion of risk since it is assumed that everyone’s individual perception of risk is different. | Risk perception is assessed regularly within the WASH literature. The approach we used draws on risk-related questions from standardised questionnaires developed by the RANAS framework (2) and Barrier Analysis (3) and adapts them for use in a group discussion. | 5 focus groups (2 with men, 3 with women) |
| Soap attributes | Participants are introduced to a set of 10 locally available soaps. These included laundry soap, scented body soap, liquid soap, and soap typically distributed by organisations. Participants are asked what criteria they use when selecting which soap to buy. They are then asked to rank the soap against these different criterion (e.g. cost, smell, duration of use, likability, perceived, most common, most typically used prior to the crisis). | It is designed to explore human-product relationships on the understanding that products which have certain attributes or ‘back-stories’ are more likely to be valued and used (4).  This method will contribute to understanding whether soap is readily available; what types of soap people have; attitudes related to soap; and how the available soap types are similar or different to soap products participants had previously been familiar with. | This method evolved from marketing research and product design (5, 6). Attribute rankings of soap have also been done in several other hygiene studies (7, 8). | 5 focus groups (2 with men, 3 with women) |
| Designing the ideal handwashing facility | Participants are introduced to a set of images of handwashing facilities from around the world. They are asked to go through each and write down the characteristics that they like or dislike about each. They are asked to review the characteristics at the end and select the three ‘must have’ features of an ideal handwashing facility. | This method assumes that the features of a product vary in importance to the user. This method is designed to identify features of a handwashing facility that are considered to be of greatest importance for encouraging use. | While there is research on participatory design processes for WASH-related products, this work often doesn’t describe the participatory process in detail. This process was modelled on a social marketing technique called This method is based upon a design research method called ‘Prune the Product Tree’(9). | 5 Focus groups (2 with men, 3 with women) |

# References

1. Quinlan M. Considerations for Collecting Freelists in the Field: Examples from Ethobotany. Field Methods. 2005;17(3):219-34.

2. Mosler H-J. A systematic approach to behavior change interventions for the water and sanitation sector in developing countries: a conceptual model, a review, and a guideline. Int J Environ Health Res. 2012;22.

3. Behaviour Change Network. Barrier Analaysis (BA). Behaviour Change Network website; 2019.

4. Odom W. Personal inventories: toward durable human-product relationships. CHI '08 Extended Abstracts on Human Factors in Computing Systems; Florence, Italy. 1358929: ACM; 2008. p. 3777-82.

5. Hanington B, Martin B. Universal Methods of Design: 100 ways to research complex problems, develop innovative ideas and design effective solutions. . MA, USA: Rockport Publishers; 2012.

6. Beebe J. Rapid assessment process: An introduction: AltaMira Press; 2001.

7. Scott B, Curtis V, Rabie T, Garbrah-Aidoo N. Health in our hands, but not in our heads: understanding hygiene motivation in Ghana. Health policy and planning. 2007;22:225-33.

8. Curtis V, Danquah LO, Aunger RV. Planned, motivated and habitual hygiene behaviour: an eleven country review. Health education research. 2009;24:655-73.

9. Hohmann L. Part 2: The Games - Prune the Product Tree. Innovation Games: Addison-Wesley Professional; 2006. p. 48-55.
