# Supplementary material for: How is hygiene behaviour affected by conflict and displacement? A qualitative case study in Northern Iraq
Source: PLoS One. 2022 Mar 3;17(3):e0264434. doi: 10.1371/journal.pone.0264434 (PMC8893612; doi:10.1371/journal.pone.0264434)
Supplement: S4 Appendix — (DOCX) [file pone.0264434.s004.docx]

Supplementary Materials – 4

Table 1: Exposure to hygiene promotion among interview participants

| **Exposure to Hygiene Promotion** | **Total**  **N = 159** | **Camp 1 N = 58** | **Camp 2 N = 49** | **Village 1 N = 18** | **Village 2**  **N = 34** |
| --- | --- | --- | --- | --- | --- |
| **Have you ever received a hygiene kit** | |  |  |  |  |
| Yes, at any point | 140 (88%) | 58 (100%) | 47 (96%) | 12 (67%) | 23 (68%) |
| Yes, in the last month | 61 (38%) | 57 (98%) | 4 (8%) | 0 (0%) | 0 (0%) |
| Never received | 19 (12%) | 0 (0%) | 2 (4%) | 6 (33%) | 11 (32%) |
| **Exposed to hygiene promotion** | |  |  |  |  |
| Yes, posters or other hygiene materials | 102 (64%) | 52 (90%) | 33 (67%) | 0 (0%) | 17 (50%) |
| Yes, attended a hygiene promotion event | 91 (57%) | 48 (83%) | 33 (67%) | 2 (11%) | 8 (24%) |
| Not exposed to hygiene materials or events | 45 (28%) | 4 (7%) | 11 (22%) | 16 (89%) | 16 (47%) |
| **Handwashing knowledge** | |  |  |  |  |
| Believe that handwashing with soap removes invisible germs from hands preventing sickness | 158 (99%) | 57 (99%) | 49 (100%) | 18 (100%) | 34 (100%) |
| Does not believe that handwashing with soap removes invisible germs from hands preventing sickness | 1 (1%) | 1 (1%) | 0 (0%) | 0 (0%) | 0 (0%) |
