# Supplementary material for: How is hygiene behaviour affected by conflict and displacement? A qualitative case study in Northern Iraq
Source: PLoS One. 2022 Mar 3;17(3):e0264434. doi: 10.1371/journal.pone.0264434 (PMC8893612; doi:10.1371/journal.pone.0264434)
Supplement: S5 Appendix — (DOCX) [file pone.0264434.s005.docx]

Supplementary materials - 5

Table 1: Summary of household observations of handwashing

| **Household observations** | **Nargizlia (n=8)** | **Sheikhan (n=6)** | **Villages (n=6)** |
| --- | --- | --- | --- |
| Total number of potential handwashing opportunities observed (based only on critical times for handwashing) | 35 | 33 | 49 |
| Number of critical times for handwashing where hands were not washed with soap | 25 (71%) | 24 (73%) | 35 (71%) |
| Number of occasions where hands were washed with any kind of soap (including non-critical times) | 38 | 39 | 27 |
| Number of occasions where hands were rinsed with water only (including non-critical times) | 34 | 37 | 25 |
| Average number of times hands were washed with soap or just rinsed during an observation period (range) | 11.7 (4-14) | 12.7 (6-18) | 8.7 (5-20) |
