# Supplementary material for: How is hygiene behaviour affected by conflict and displacement? A qualitative case study in Northern Iraq
Source: PLoS One. 2022 Mar 3;17(3):e0264434. doi: 10.1371/journal.pone.0264434 (PMC8893612; doi:10.1371/journal.pone.0264434)
Supplement: S6 Appendix — (DOCX) [file pone.0264434.s006.docx]

Supplementary Materials - 6

Table 1: Handwashing facility design factors that FGD participants thought would be most likely to increase their handwashing behaviour

| **What aspects of the handwashing facility design would be most likely to increase your handwashing behaviour?** | | | | | |
| --- | --- | --- | --- | --- | --- |
| **Nargizlia Men’s FGD** | **Nargizlia Women’s FGD** | **Sheikhan Men’s FGD** | **Sheikhan Women’s FGD** | **Villages Men’s FGD** | **Villages Women’s FGD** |
| Make the facility private/not shared | A mirror above the facility | A mirror above the facility | Sink should be outside the toilet | A mirror above the facility | Liquid Soap |
| Make the facility comfortable and enjoyable to use | Make the facility private/not shared | A sink to catch waste water | Child friendly / a design that motivates children to wash hands | Easy to keep clean | A sink to catch waste water |
| Minimise water use | Liquid soap | Reminders/ nudges to cue handwashing | Liquid soap | Minimise water use | A mirror above the facility |
| Something to prevent the soap from being taken | Easy to keep clean | Child friendly / a design that motivates children to wash hands | A sink to catch waste water | Accessible for people with disabilities | Additional public handwashing facilities throughout the village |
