# Supplementary material for: How is hygiene behaviour affected by conflict and displacement? A qualitative case study in Northern Iraq
Source: PLoS One. 2022 Mar 3;17(3):e0264434. doi: 10.1371/journal.pone.0264434 (PMC8893612; doi:10.1371/journal.pone.0264434)
Supplement: S7 Appendix — (DOCX) [file pone.0264434.s007.docx]

|  | **Nargizlia Camp** | | **Sheikhan Camp** | | **Villages** | |
| --- | --- | --- | --- | --- | --- | --- |
|  | **Female FGD** | **Male FGD** | **Female FGD** | **Male FGD** | **Female FGD** | **Male FGD** |
| **What is the likelihood of your child getting diarrhoea in the next 6 months** | Moderate | Moderate | Moderate | Unlikely | Unlikely | Unlikely |
| **If your child got diarrhoea how badly would it affect your life?** | Lots of bad effects | Lots of bad effects | Some bad effects | Some bad effects | Lots of bad effects | Some bad effects |
| **If your child got diarrhoeal how likely is it that it could result in death?** | Moderate | Moderate | Moderate | Unlikely | Unlikely | Unlikely |
| **What is the likelihood of you or your child getting diarrhoea now that you live here (e.g. in a camp), compared to where you lived before?** | More likely | More likely | More likely | More likely | Equal likelihood | Equal likelihood |
| **How worried are you about diarrhoea?** | Major concern | Major concern | Minor concern | Minor concern | Minor concern | Minor concern |
| **How easy is it to prevent diarrhoea?** | Difficult | Difficult | Difficult | Easy | Easy | Moderate |
| **Do you feel like you have the ability to prevent diarrhoea?** | Able to prevent | Unable to prevent | Unable to prevent | Able to prevent | Able to prevent | Some ability to prevent |
| **How important is diarrhoea to other people in this camp/village?** | Very important | Moderate importance | Very important | Moderate importance | Moderate importance | Moderate importance |

Supplementary Materials – 7

Table 1: Heat map of scaled FGD responses to diarrhoeal risk related questions
