# Supplementary material for: How is hygiene behaviour affected by conflict and displacement? A qualitative case study in Northern Iraq
Source: PLoS One. 2022 Mar 3;17(3):e0264434. doi: 10.1371/journal.pone.0264434 (PMC8893612; doi:10.1371/journal.pone.0264434)
Supplement: Attachment — Submitted filename: Review comments for Plos One.docx [file pone.0264434.s008.docx]

**Review comments**

**Manuscript Number:** PONE-D-21-15601

**Full Title:**

How does hygiene behaviour change over the course of displacement? A qualitative case study in Iraq and Kurdistan.

**Short Title:**

How does hygiene behaviour change over the course of displacement?

**Comments**

Firstly, I would like to congratulate the authors for this good piece of work that is very important in the field of hand hygiene. The findings will add on the existing knowledge and may be applied across the globe in the displaced society. With the current state of the devastating Covid-19, its worthy to consider publishing the article in Plos One so as to be accessed by the wider community. Hand hygiene is one of the intervention much touted for Covid-19 prevention.

**Abstract**

The abstract reads well. However, the authors have indicated that the study took place in two countries (Irag and Kurdistan), as it appears in the title of their study. Yet the authors have only put Iraq in the key words.

I suggest that they also add Kurdistan or remove Iraq from the key words. Or if its one country, ten that has to be made clear throughout the study. It is confusing that Kurdistan appears as a country in some other instance but it also appears as part of Iraq. This has to be clarified

**Introduction**

Page 3, line 69; authors have mentioned that the setting of the study is in Northern Iraq. Yet in the title authors talk of two settings, thus Iraq and Kurdistan. Furthermore, in the abstract, authors have just mentioned of Iraq, and not Northern Iraq as is the case in the introduction.

I suggest that author need to be consistent I writing Iraq versus Northern Iraq. And also consistency on the setting of the study.

Lines 67 to 68, authors have introduced holistic behavioural framework and a range of participatory methods. This has been introduced suddenly and left in suspense.

I suggest that authors provide a little more information about the holistic behavioural framework in the introduction section. What it is and its relevance/applicability to this study

**Methods**

Page 5, lines 93-101; Authors are talking about the framework used in the study. They have also talked about data collection methods; this is recommendable. However, I suggest that author should first talk about the framework they have claimed to use as written in the introduction (the holistic behavioural framework). Then they should link it with the framework written in this section. There is a missing link between the framework the authors have written in introduction on lines 67-68 and the framework with the constructs written in this section (lines 93-101)

On page 5, line 100, authors have written (SM1 1) and this seem to appear for the first time. I suggest that authors write it in full the first time before writing the abbreviation

**Page 5 under “observations”;**

Authors need to indicate those who did the observations, in terms of their expertise, qualifications, how many they were, how they ensured consistency between/among observers (validity). If they are the same who are mentioned on page 7 under data collection, (line 136), then they should first appear here.

**Data collection, management and analysis**

Line 135, it s indicated that the initials are removed for review. But then immediately, in the next sentence 136) the initials appear with their nationality. I suggest they put the initials since they are already appearing on the second line for consistency.

Also just as I mentioned above, the ones mentioned here to have collected data, and if they are the one referred to have made the observations, then they must appear under observation on page 5 first to avoid the audience wondering who these observers or data collectors are, and just to find their initials later in the document.

**Results**

Results are well written and clear. There is a good logical flow of presentation of the results.

**Discussion**

The interpretation of results is sufficiently substantiated by data.

There is coherence between qualitative data sources, collection, analysis and interpretation.

**Limitations of the study**

Observation in relation to socially acceptable behaviour. This is because participants were observed practicing hand hygiene, and they knew they were video recorded. This limitations must come out strongly in their study.
